# Microstructure Evolution and Properties Induced by Multi-Pass Drawing of Graphene/Copper Nanocomposite

**DOI:** 10.3390/nano12050807

**Published:** 2022-02-28

**Authors:** Miao Wang, Jie Sheng, Changsheng Xing, Gang Wang, Yuanpei Duan, Lidong Wang

**Affiliations:** 1 School of Materials Science and Engineering, Anhui Polytechnic University, Wuhu 241000, China; wangmiao@ahpu.edu.cn (M.W.); gangwang@ahpu.edu.cn (G.W.); dyp@ahpu.edu.cn (Y.D.); 2 Laboratory for Space Environment and Physical Science, Research Center of Basic Space Science, Harbin Institute of Technology, Harbin 150001, China; 3 School of Materials Science and Engineering, Harbin Institute of Technology, Harbin 150001, China; xingcs7@163.com

**Keywords:** graphene/Cu composite, cold drawing, microstructure evolution, tensile properties, electrical conductivity

## Abstract

The influence of multi-pass cold drawing on the evolution of microstructure, texture, and properties of Cu matrix composite, reinforced by in situ grown graphene, has been systematically investigated. Under continuous and severe plastic deformation, the grains in the composite were continuously refined to nanoscale. In addition, graphene in the composite could be gradually refined, exfoliated, and redispersed. Interestingly, dynamic recrystallization of the composite was formed after 80% drawing reduction and its formation mechanism was discussed. The texture of the as-drawn composite comprised a mixture of fiber textures with dominated <111> and minor <100> orientation after 99.7% severe drawing reduction. The tensile properties and electrical conductivity of the as-drawn composites were also investigated. This work provides a better guideline on the plastic deformation behavior of the advanced graphene/metal nanocomposite.

## 1. Introduction

Metal matrix composites reinforced by graphene (Gr/MMCs) have recently attracted considerable attention and researchers strive to improve its structural or functional properties [1,2,3,4,5]. The dispersion of graphene in metal matrix and the interfacial bonding between graphene and metal matrix are the two basic challenges for fabricating Gr/MMCs with high performance [6]. On the one hand, ball milling [4], molecular-level mixing [7,8], semi-powder metallurgy [9], and in situ grown method [2,10,11,12] have been exploited to disperse graphene in metal matrix. Of note, the in situ grown method has attracted significant attention due to the effective dispersion of graphene in matrix. On the other hand, the rational design of interfacial microstructure between graphene and metal matrix, aiming at fully developing the remarkable properties of graphene, has also attracted wide attention [12,13,14]. Among the Gr/MMCs, graphene/Cu composites are the most widely investigated due to the variety of their preparation methods [3,5,7,8,9,10,11,12]. In particular, the in situ grown method is very suitable for the fabrication of graphene/Cu composites due to the surface-catalyzed ability of Cu [15]. Additionally, there are various investigations regarding the properties of graphene/Cu composites. For instance, the mechanical, electrical [16,17,18,19,20,21,22], and thermal properties [23,24] of graphene/Cu composites are most commonly studied.

As a result, the plastic deformation of composites is the next step. In addition, it is crucial to the realization of the transformation from blank materials to semi-manufactured products. Commonly, several plastic deformation methods, such as extrusion [25], rolling [26], and drawing [27] are used to fabricate the semi-products or products of the composites (e.g., rods, sheets, and wires). For instance, hot rolling is often used for the formation of metal plate materials. In recent years, the hot rolling behavior of graphene or carbon nanotube reinforced metal composites has been extensively studied [28,29]. As recently systematically reported by our research group, the hot rolling behavior of Cu nanocomposite reinforced by graphene indicates that the hot rolling process has a significant effect on both the plastic deformation of matrix and graphene, which affects the properties of composites [30]. Another plastic deformation technique, cold drawing, has recently been gradually applied to fabricate advanced graphene/metal wires using its severe plastic deformation [27,31]. For instance, Li et al. [27] studied a graphene/Al composite wire prepared by powder metallurgy and multi-pass cold drawing, and found that the dispersion of graphene and mechanical properties of the composite could be improved during the cold drawing. Li et al. [31] obtained Cu/graphene composites with high mechanical and electrical properties using cold drawing. The authors found that the original agglomerated graphene could be redispersed, and finally formed a network structure. Zhao et al. [32] fabricated a graphene/Cu composite wire with a synergistic method that consists of the growth of graphene on Cu wire, through twisting and drawing of the wires. The authors found that the ampacity of the composite wire could be significantly improved by introducing graphene. Although the drawing deformation has been successfully used in Gr/MMCs, the investigation of its drawing behavior is still inadequate [33]. To our knowledge, graphene has excellent mechanical properties [15]. Therefore, during the plastic deformation, the microstructure and properties of composite can be markedly influenced by the incorporated graphene. Additionally, the size, morphology, and dispersion of graphene can be affected due to the deformation of matrix [30,34]. As a result, it is significant to systematically investigate the evolution of microstructure and properties of Gr/MMCs during the drawing process.

Herein, graphene/Cu composite wires were prepared by in situ grown graphene on Cu powders, spark plasma sintering (SPS), hot-extrusion, and multi-pass cold drawing. During the process of cold drawing, grains in the composite could be continuously refined to nanoscale and the homogeneity of microstructure was improved. Moreover, graphene in the composite was found to be gradually refined, exfoliated, and redispersed. Interestingly, after severe plastic deformation, the recrystallization of composites was formed. The underlying deformation mechanism was discussed. As a result, the composites showed an improvement of mechanical properties and electrical conductivity after cold drawing. This work provides a deep understanding of the plastic deformation process of Gr/MMCs and can be a guide for the preparation of Gr/MMCs with high performance.

## 2. Experimental Section

### 2.1. Synthesis of Graphene on Cu Particles via an In Situ Method

Commercial flake Cu powders (purity of 99.9%) with a diameter of 30~50 μm and solid molecular naphthol (analytical reagent (AR)) were used for the raw Cu matrix and carbon source, respectively. The typical growth process of graphene, which was systematically studied in our recent investigation [11], can be briefly described as follows: In the first step, naphthol (0.1 wt%) and Cu powders were added in ethanol (AR) with constant stirring and the solution was sonicated for 20 min. Then, the ethanol was rapidly removed using a rotary evaporator at 130 °C and naphthol-coated Cu powders were obtained. Second, naphthol-coated Cu powders were graphitized at 800 °C for 10 min using a quartz tube furnace under H_2_ (17 mL/min)/Ar (83 mL/min) mixture. Thereafter, in situ grown graphene on Cu composite powders could be achieved.

### 2.2. Composite Fabrication

The as-prepared composite powders were first sintered using SPS (SP-250, Germany) (pressure of 40 MPa, 950 °C for 30 min). Then, a rod (Φ9.5 × 40 mm) was cut from the as-sintered composite. Subsequently, the rod was extruded at 750 °C (extrusion ratio of 7.4:1). The as-extruded composite (diameter of 3.5 mm) was drawn by the multi-pass process at room temperature and the reduction of each single pass was less than 10%. Finally, the as-drawn composites with different diameters were obtained. The area reduction is calculated as the drawing reduction, and Table 1 presents the drawing reduction of the composites with different diameters.

### 2.3. Characterizations

The microstructure was carried on scanning electron microscopy (SEM, Nanolab-600i, Hillsboro, OR, USA) and transmission electron microscope (TEM, Talos, F-200X, Portland, OR, USA). Prior to the SEM investigation of individual graphene in graphene/Cu composite powders, the composite powders were first etched by HNO_3_ (5 wt%) to remove Cu. The composites for TEM analysis were prepared by several steps of mechanical grinding to obtain a foil (thickness of 50~60 μm), and ion thinning technique. The C content of graphene in Cu matrix is tested by carbon and sulfur analyzer (CS901B, Zhengzhou, China). The room temperature tensile properties of block composites were studied by an Instron-1186 tensile testing machine. In addition, the tensile speed, gauge length, and gauge width were 0.5 mm/min, 15 and 2 mm, respectively. The tensile properties of as-drawn composites (length of 20 mm) were measured on a tensile machine (HRJ, WDW-1D, Dongguan Hongtuo Instrument, Dongguan, China) with a speed of 0.5 mm/min. The resistance of the samples was tested on a low resistance instrument based on the four-wire measurement method (CS2512, Allwin Instrument, Nanjing, China). The electron backscatter diffraction (EBSD, ZEISS-SUPRA55, Oberkochen, Germany) of the composites was carried to investigate the microstructure of the composite. The EBSD system is HKL Nordlys (Oxford Instrument, Abingdon, UK), and the data processing software is Channel5. The samples for EBSD investigation were prepared by the cross section ion polishing instrument (IB-09020CP, Tokyo, Japan). For SEM and EBSD test, the observation positions of as-extruded and as-drawn composites were investigated on the transverse direction–normal direction (TD–ND) plane.

## 3. Results and Discussion

### 3.1. Characterizations of As-Grown Graphene on Cu Powders

**Figure 1:**
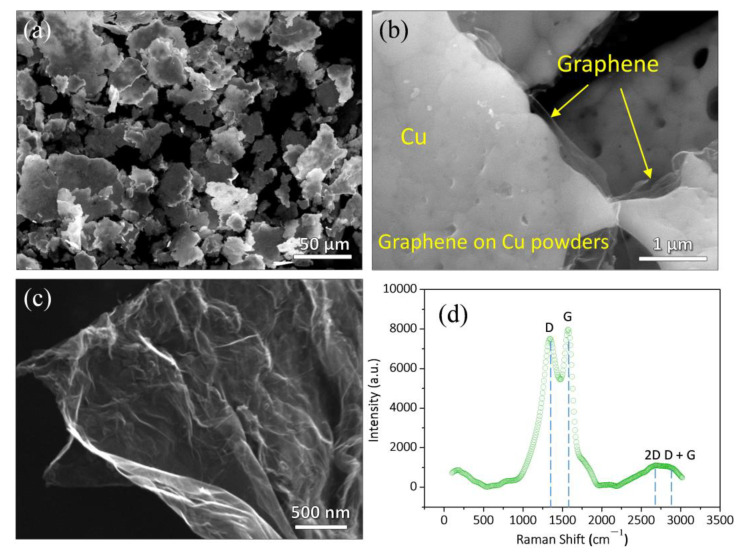
SEM morphology of (**a**) initial flake Cu powders, (**b**) as-grown graphene on Cu powders, and (**c**) graphene after etching Cu, (**d**) Raman curve of as-grown graphene (magnifications for (**a**–**c**) are ×1000, ×60,000, and ×50,000, respectively).

In Figure 1a, the SEM morphology demonstrates that the size of initial flake Cu powders is about 30~50 μm. Of note, the main reason for the use of flake Cu powders rather than granular Cu powders is that flake Cu powders have a larger specific surface area, which can adsorb more carbon source, and thus grow more graphene compared with the particles [10,11]. In Figure 1b, it can be seen that the as-grown graphene is highly transparent, with high surface quality and typical wrinkles. Figure 1c presents a typical morphology of the as-grown graphene after etching Cu powders, and typical wrinkles on the graphene can be clearly found. In Figure 1d, Raman spectra reveals the typical D band (1357 cm^−1^), G band (1603 cm^−1^), and 2D band (2300~3000 cm^−1^) of the graphene. The I_D_/I_G_ (intensity ratio of D to G band) of the as-grown graphene is calculated as 0.89, indicating a low defect density of the graphene [35]. All of the aforementioned analyses indicate that high quality graphene has been successfully fabricated.

### 3.2. Microstructure of As-Sintered Composite and As-Extruded Composite

**Figure 2:**
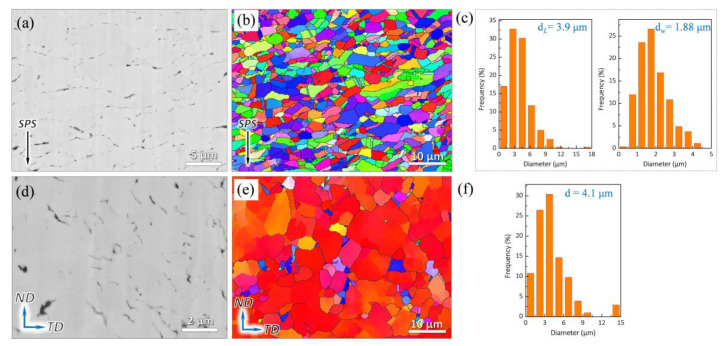
SEM-BSE images of (**a**) as-sintered and (**d**) as-extruded composites. IPF maps of (**b**) as-sintered and (**e**) as-extruded composites, the arrows in (**a**,**b**) show the direction of pressure of SPS. (**c**) Grain size of the as-sintered composite. The width and length of grains are expressed as d*_W_* and d*_L_*, respectively. (**f**) Grain size of the as-extruded composite (magnifications for (**a**,**d**) are ×10,000 and ×20,000, respectively).

The dispersibility of graphene is one of the most challenging difficulties for the preparation of composite. Nevertheless, this problem can be easily solved by the graphene in situ growth method. Therefore, the as-obtained composites can be a model material for the study of plastic deformation mechanism. As shown in Figure 2a, the typical SEM-BSE image of the as-sintered composite reveals that graphene sheets, corresponding to the black strips, can be clearly found to be uniformly dispersed in the composite. Additionally, the arrangement direction of graphene tends to be perpendicular to the pressure direction of sintering, which is attributed to the directional alignment of flake Cu powders during the sintering process. Figure 2b shows the IPF map of the as-sintered composite, and the arrangement of strip Cu grains is consistent with Figure 2a. Furthermore, Figure 2c presents the grain size in Figure 2b, and the width (d*_W_*) and length (d*_L_*) of the grains are counted as 1.88 and 3.9 μm, respectively. Nevertheless, Figure 2d demonstrates that the dispersion of graphene in as-extruded composite is random. The refinement and exfoliation of graphene, compared with the as-sintered composite, can be clearly found in the as-extruded composite. On the one hand, the low plasticity of graphene compared with the Cu can lead to the fracture and refinement of graphene during the extrusion deformation. In addition, due to the weak van der Waals force in its interlayer, graphene can be easily exfoliated by the shear stress existing in the hot-extruded process [36]. In Figure 2e, the IPF map of the as-extruded composite presents the morphology of grains (diameter of 4.1 μm), which changes from strip to irregular grains (see Figure 2f).

**Figure 3:**
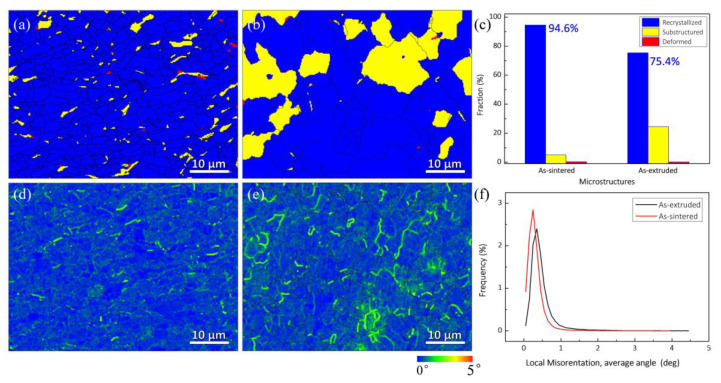
Recrystallized, substructured, and deformed grains in (**a**) as-sintered and (**b**) as-extruded composites, (**c**) statistics of grains in (**a**,**b**). KAM maps of (**d**) as-sintered and (**e**) as-extruded composites, (**f**) KAM values, corresponding to (**d**,**e**).

Figure 3a,b exhibits three types of grains, namely recrystallized, substructured, and deformed grains, as well as the distribution in the as-sintered and as-extruded composites, respectively. Recrystallized grains (94.6%, see Figure 3c) dominate in the as-sintered composite due to the high temperature and long holding time of sintering. Following the hot extrusion, the percentage of the recrystallized grains drops to 75.4%. To further investigate the microstructure of the composites, kernel average misorientation (KAM) was carried out from EBSD. KAM is often used to analyze the distribution of microstrain in crystals, which is related to the degree of microstrain and dislocation density [37,38]. Figure 3d,e presents the KAM maps of the as-sintered and as-extruded composites, respectively. It can be seen that the microstrain in both composites is relatively small, indicating a low dislocation density. Furthermore, Figure 3e shows the KAM values of the composites and quantitatively describes the variation of KAM values. Herein, it can be seen that the peak KAM values of the two composites are distributed at low orientation angle, indicating a low dislocation density in both as-sintered and as-extruded composites.

**Figure 4:**
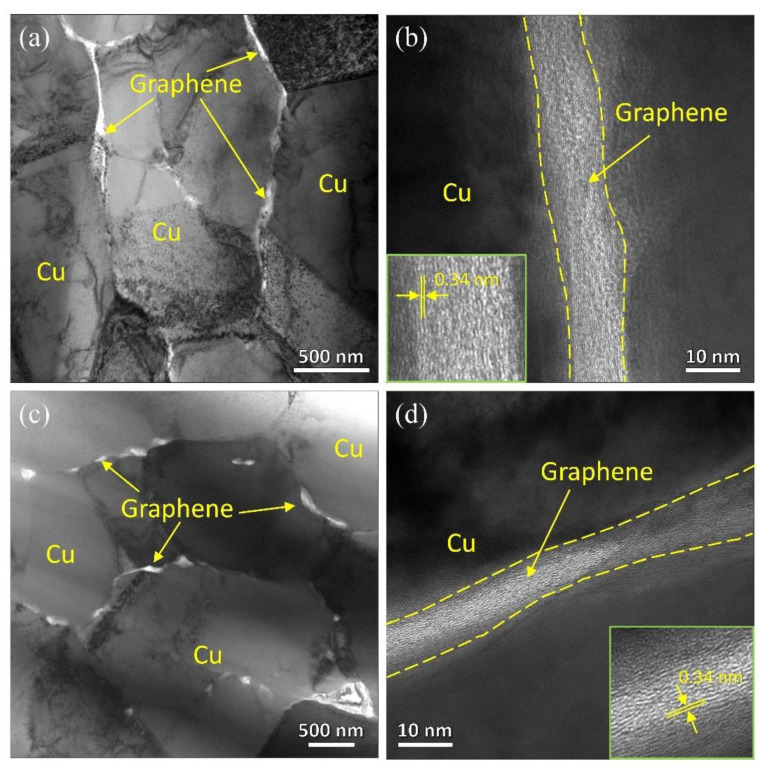
TEM images of the (**a**) as-sintered and (**c**) as-extruded composites, HRTEM images of the (**b**) as-sintered and (**d**) as-extruded composites.

Figure 4 presents the TEM investigation of the as-sintered and as-extruded composites. In Figure 4a,c, the dislocation density in the two composites is low, corresponding to the KAM maps in Figure 3. Graphene sheets are found to be homogeneously distributed and located at grain boundary in matrix. Additionally, the refinement and exfoliation of graphene can be clearly found after hot extrusion, which is consistent with the SEM-BSE observation in Figure 2. Furthermore, Figure 4b,d exhibits the typical HRTEM microstructure of graphene, which is confirmed by the interlayer spacing of about 0.34 nm [39]. The microscopic holes or impurity phases cannot be found at the interface of composite, indicating a well interfacial bonding.

### 3.3. Evolution of Graphene during Cold Drawing

**Figure 5:**
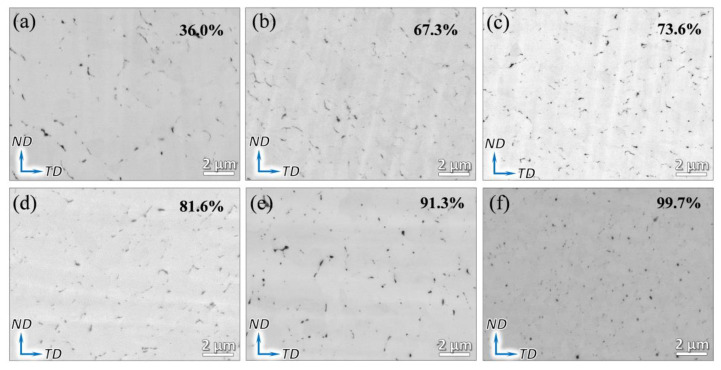
SEM-BSE images of the as-drawn composite with different drawing reductions. (**a**) 36.0%, (**b**) 67.3%, (**c**) 73.6%, (**d**) 81.6%, (**e**) 91.3%, and (**f**) 99.7%.

Typical SEM-BSE images of the as-drawn composites are shown in Figure 5a–f. Overall, with the drawing reduction increase from 36.0% to 99.7%, it can be found that graphene is homogeneously dispersed in matrix, which can be first attributed to the in situ graphene uniformly grown on Cu powders. Additionally, graphene can be redispersed during the drawing deformation [27]. Furthermore, the refinement and exfoliation of graphene can be clearly found with the increasing drawing reductions. Similarly, in the aforementioned discussion regarding the extrusion process, graphene can be easily fractured and refined during the drawing deformation due to the great discrepancy in plasticity between graphene and Cu. Therefore, the tensile or compressive stress resulting from drawing can refine the graphene to small pieces. Thick graphene or graphite can be in situ exfoliated into thin graphene during the plastic deformation process, which has been reported in recent investigations [30,36,40]. The shear strain induced by deformation is considered to be the inducement for the exfoliation of graphene. Similarly, under the shear strain existing in the drawing [41], graphene can be exfoliated. Of note, only thick graphene can be clearly observed under the backscattered electron mode, while thin graphene only presents a low contrast and is difficult to be observed (see Figure 5). XRD patterns of the powders and composites with different drawing reductions have also been investigated (Figure S1, see Supporting Information). It can be found that all of the XRD curves present a pure phase of Cu. In addition, no graphene peak can be found since the content of graphene in Cu matrix is very low (only 0.3 vol%), which is tested by a carbon and sulfur analyzer.

### 3.4. Microstructure Evolution of Cu Matrix during Drawing

**Figure 6:**
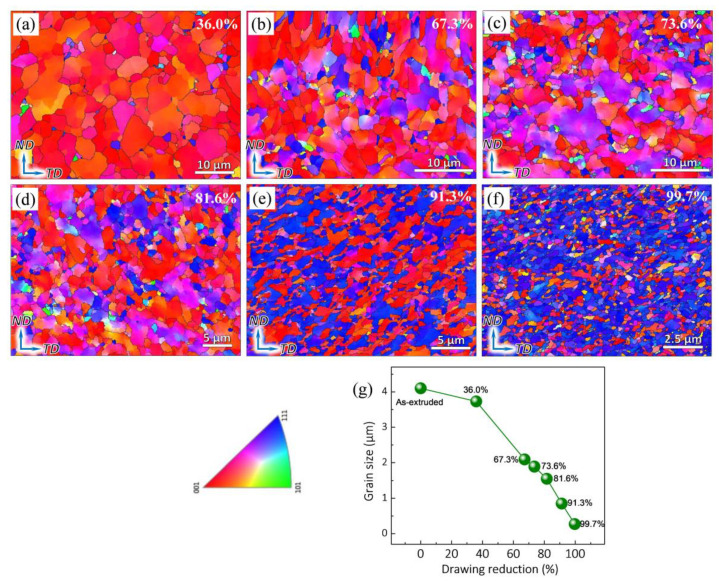
IPF images of the as-drawn composites with different drawing reductions. (**a**) 36.0%, (**b**) 67.3%, (**c**) 73.6%, (**d**) 81.6%, (**e**) 91.3%, and (**f**) 99.7%. (**g**) The variation of grain size with different drawing reductions.

EBSD was used to study the microstructure evolution of the composites during the drawing process, including the evolution of grain size, grain boundaries (GBs), recrystallization, microstrain, and texture. Figure 6a–f presents the IPF maps on the ND–TD plane of the composites with different drawing reductions. The grain size of the as-drawn composites is continuously found to be 3.73, 2.09, 1.89, 1.55, 0.85, and 0.27 μm, corresponding to the drawing reductions of 36.0%, 67.3%, 73.6%, 81.6%, 91.3%, and 99.7%, respectively (see Figure 6g). In addition, it can be found that, with the increasing drawing reductions, the microstructure homogeneity of the composites can be evidently improved.

**Figure 7:**
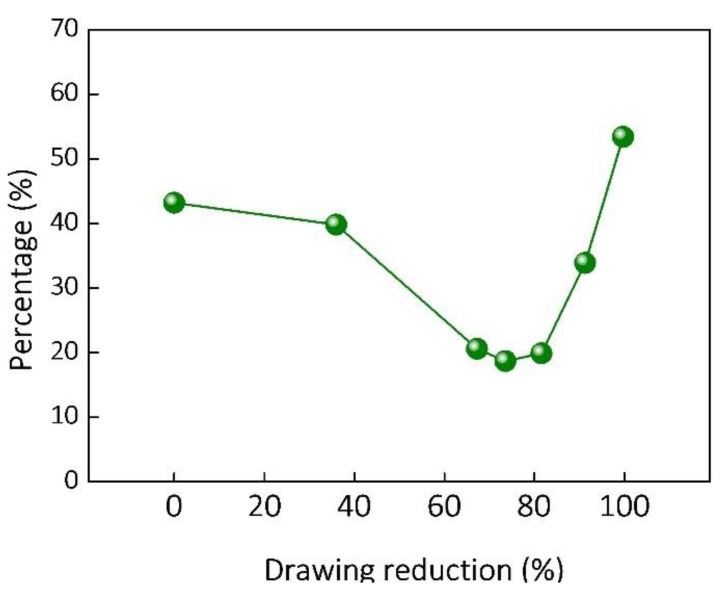
Percentage of HAGBs of the composites with different drawing reductions.

To investigate the characteristics of GBs in the composites, the type and misorentation angle distribution of GBs are presented in Figure S2 (see Supporting information). It can be observed that GBs in the as-sintered composites are dominated by high-angle GBs (HAGBs) and twinning boundaries (TBs) (see Figure S2a), which can be attributed to the condition of high temperature sintering. Following the deformation by extrusion and drawing, the fraction of TBs decreases evidently and the GBs are gradually dominated by low-angle GBs (LAGBs) and HAGBs. Figure 7 shows that the percentage of HAGBs first decreases with the increasing drawing reductions from 36.0% to 67.3%, and then stabilizes from 67.3% to 81.6%. When the drawing reduction further increases from 81.6% to 99.7%, the percentage of HAGBs increases rapidly and its proportion exceeds 50% when the drawing reduction increases to 99.7%. The evolution of GBs of the composite is associated with the formation of recrystallized and subgrains in the composites [42]. The related mechanism will be discussed in the following section.

**Figure 8:**
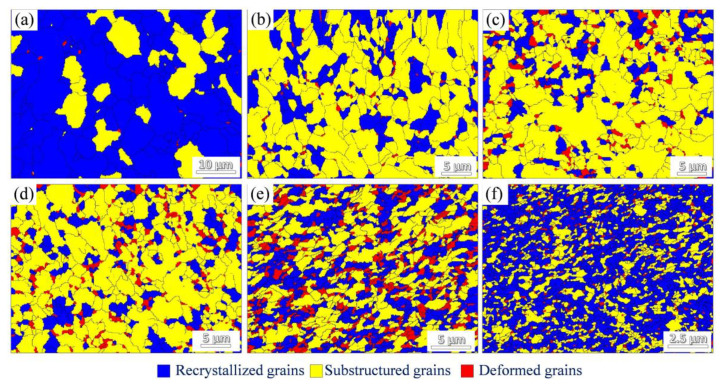
The distribution of recrystallized, substructured, and deformed grains of the as-drawn composites with different drawing reductions: (**a**) 36.0%, (**b**) 67.3%, (**c**) 73.6%, (**d**) 81.6%, (**e**) 91.3%, and (**f**) 99.7%.

**Figure 9:**
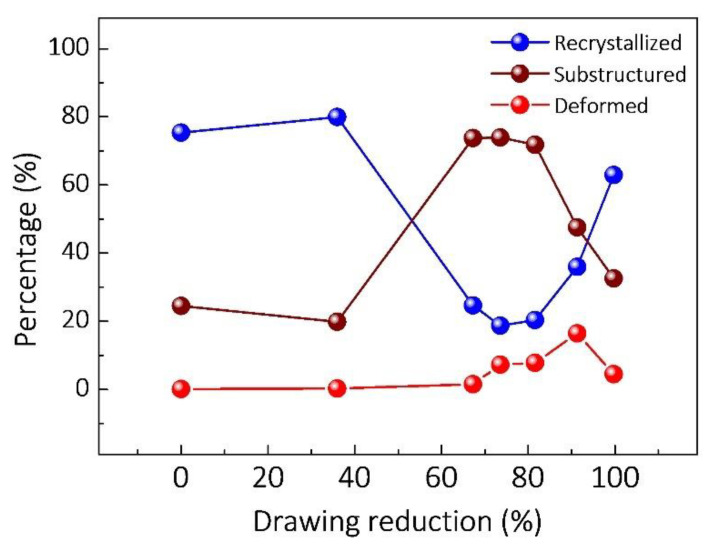
The percentage of recrystallized, substructured, and deformed grains of the as-drawn composites with different drawing reductions.

Furthermore, Figure 8 reveals the microstructure of the composite during the drawing process. In addition, the recrystallized, substructured, and deformed grains of the composites are marked. According to the algorithm in Channel5 software, the discrimination of different grain types is based on the internal average misorientation angle (IAMA). Substructured grains are the grains with IAMA under 1° and the misorientation between the subgrains is above 2°. Deformed grains are the grains with IAMA above 1°. The remaining grains are recrystallized grains. Figure 9 shows the quantitative analysis of grains fraction, corresponding to Figure 8. It can be found that the variation trend of the fraction of recrystallized grains is similar to the HAGBs in Figure 7. In particular, the microstructure is still dominated by recrystallized grains at a small drawing reduction of 36%, which can be attributed to the high fraction recrystallized grains reserved from the as-extruded composite. Then, with the increasing drawing reductions, the recrystallized grains first decrease, then stabilize, and finally increase. The fraction of recrystallized grains is higher than 60% when the drawing reduction is 99.7%, which reveals that the dynamic recrystallization grains are formed under the condition of severe drawing deformation.

Since the composites are drawn at room temperature, how does the mechanism of the dynamic recrystallization form? The underlying reasons are discussed as follows: (i) Initially, for the matrix, the GBs in the initial as-extruded composite are dominated by HAGBs (see Figure 3c). The reason is that the dislocation movement proceeds easily at high extrusion temperature, which can promote the formation of LAGBs. Then, the LAGBs can further absorb dislocations and transform into HAGBs [43,44], leading to the high percentage of recrystallized grains. With the process of drawing deformation at a low reduction (~67.3%), the multiplication and movement of dislocations can proceed and form a substructure. Therefore, the proportion of HAGBs decreases, corresponding to the decrement of recrystallized grains. With the increase of drawing reductions from 67.3% to 81.6%, the formation of substructure can also continuously absorb dislocations, and then gradually transform into HAGBs, leading to a dynamic equilibrium of HAGBs and recrystallization grains. Finally, the substructured grains can transform into recrystallized grains by continuously absorbing dislocations [45]. (ii) For the graphene, the uncoordinated plastic deformation ability between Cu and graphene can contribute to the production of deformation heterogeneities regions in matrix. These regions have high storage energy and can accelerate the formation of recrystallization [46]. Moreover, the homogenization of deformation substructure (see Figure 8) resulting from the uniform distribution of graphene can be a preferential point for the formation of recrystallization grains [47]. Additionally, the introduction of graphene increases the number of interfaces in the composite, which can accelerate the dislocation annihilation and promote the formation of recrystallization [48]. To summarize, the recrystallization of composites under severe deformation is affected by a synergistic effect from the matrix and graphene.

**Figure 10:**
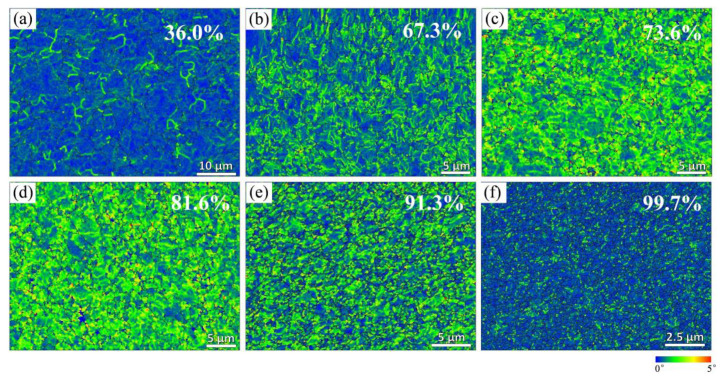
KAM maps of the as-drawn composites with different drawing reductions: (**a**) 36.0%, (**b**) 67.3%, (**c**) 73.6%, (**d**) 81.6%, (**e**) 91.3%, and (**f**) 99.7%.

**Figure 11:**
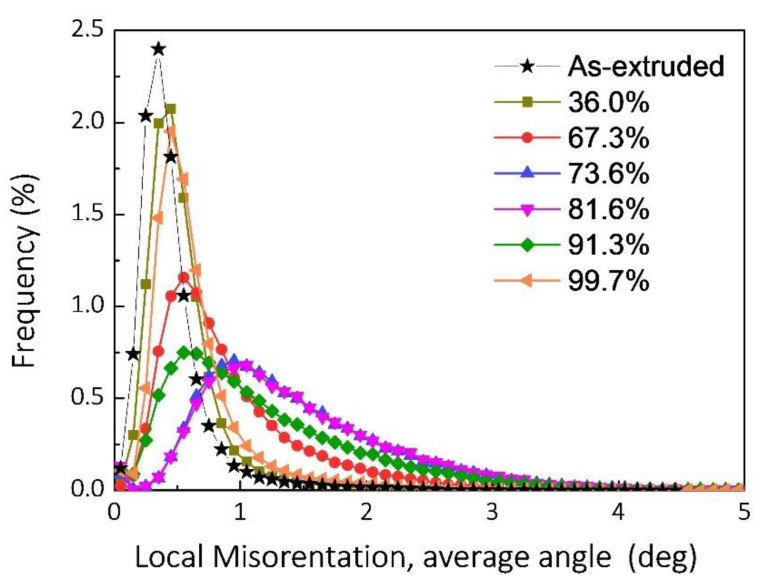
KAM values of the composites with different deformation reductions.

Figure 10 shows the KAM maps of the as-drawn composites with the increasing drawing reductions. Overall, the microstrain in the as-drawn composites increases first and then decreases with the increasing drawing reductions. Of note, the microstrain distribution in composite is uniform and no evident strain concentration can be found, which benefits from the uniformly distributed graphene. Figure 11 quantificationally evaluates the KAM values, corresponding to the KAM maps in Figure 8. It can be clearly seen that the variation trend of the KAM peak values is consistent with the observation from the microstrain in the composite. It is commonly known that the KAM curve with the high peak value indicates the high dislocation density [49,50]. Therefore, it can be clearly found that the variation of dislocation density is consistent with the variation tendency of the recrystallization of composites. Therefore, the evolution of recrystallization process of the composites can be confirmed more directly by the aforementioned KAM observation.

**Figure 12:**
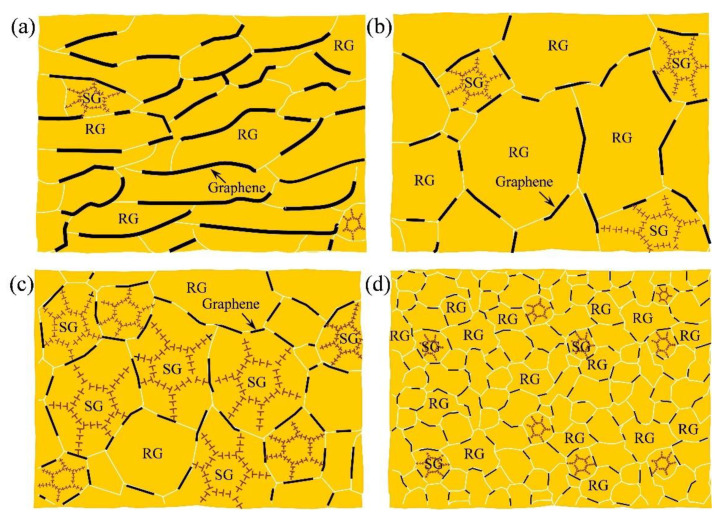
An illustration scheme of the evolution of graphene and matrix during the drawing process (on the ND–TD plane). (**a**) As-sintered composite, (**b**) as-extruded composite, (**c**) as-drawn composite dominated by substructured grains (SG) and (**d**) as-drawn composite dominated by recrystallized grains (RG).

Figure 12 is an illustration scheme for the evolution of graphene and matrix during the drawing process. As shown in Figure 12a, original thick graphene sheets are distributed at the interface in the as-sintered composite. Then, the refinement and exfoliation of graphene can be gradually formed with the increasing drawing reductions. The detail mechanism is discussed in Section 3.3. As a result, homogeneously distributed graphene sheets with small pieces and thickness can be obtained in the final composite (see Figure 12d). For the matrix, the grains morphology change from original strip grains to equiaxed grains, corresponding to the as-sintered and as-extruded (or as-drawn) composites, respectively. Additionally, after severe drawing reduction, the recrystallization of composites can be formed, which is affected by a synergistic effect of matrix and graphene (see the discussion in Section 3.4).

### 3.5. Evolution of Texture during Drawing

**Figure 13:**
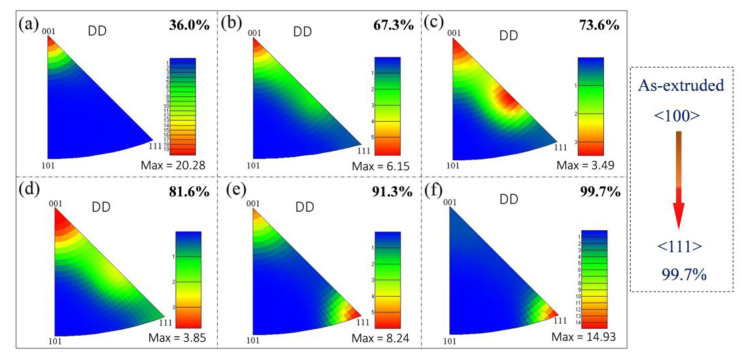
Inverse pole figures of the composites with different drawing reductions: (**a**) 36.0%, (**b**) 67.3%, (**c**) 73.6%, (**d**) 81.6%, (**e**) 91.3%, (**f**) 99.7%.

**Figure 14:**
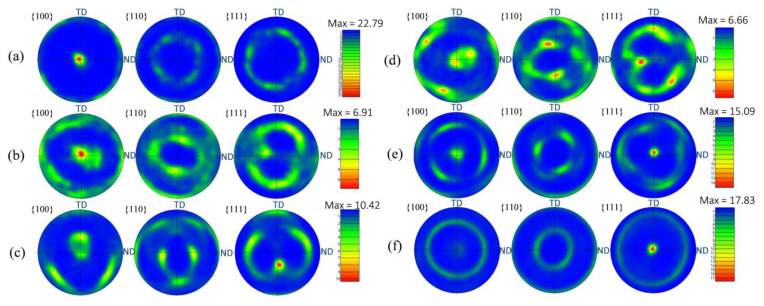
Pole figures of the composites with different drawing reductions: (**a**) 36.0%, (**b**) 67.3%, (**c**) 73.6%, (**d**) 81.6%, (**e**) 91.3%, (**f**) 99.7%.

During the drawing deformation of FCC metals, the <100> and <111> orientation will parallel to the axis direction, and form a mixture of <100> and <111> fiber textures. Among them, the <111> fiber texture is considered to be a stable texture, while the <100> fiber texture is a transitional texture. In addition, the <100> fiber texture is mainly attributed to the recrystallization during deformation [51,52]. As shown in Figure S3, a strong and single <100> fiber texture is formed in the as-extruded composite, which can be due to the recrystallization at high extrusion temperature. Figure 13 reveals the inverse pole figures of the composites with the increasing drawing reductions from 36.0% to 99.7%. It can be found that the types of fiber textures are <100> (see Figure 13a), <100> and <112> (see Figure 13c) to <111> and <100> (see Figure 13e), corresponding to the drawing reduction of 36.0%, 73.6%, and 91.3%, respectively. Finally, a mixture of fiber textures with dominated <111> and minor <100> fiber textures is formed when the drawing reduction is 99.7% (see Figure 13f). The texture results reveal that the grains with <100> orientation gradually decrease, while the grains with <111> orientation gradually increase, indicating that the orientation of grains gradually rotates from <100> to <111> orientation during the drawing process. In addition, the final texture induced by the severe drawing reduction is consistent with these results in the drawing deformation of pure Cu, indicating that the incorporation of graphene does not change the type of texture of Cu [53,54]. Although the dynamic recrystallization occurs in the composites after severe plastic deformation, the nucleation and growth of grains are not formed. Therefore, the texture of the as-drawn composite can still retain the fiber texture generated from the drawing deformation of Cu. Figure 14 exhibits the pole figures of the composites with different drawing reductions, which is consistent with the results obtained in Figure 13.

### 3.6. Tensile Properties and Electric Conductivity of Composites

**Figure 15:**
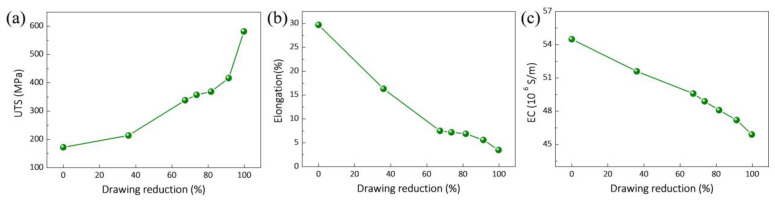
Tensile properties and electric properties of composites with different drawing reductions: (**a**) UTS, (**b**) elongation and (**c**) electrical conductivity (EC).

Figure 15 presents the tensile properties and electric properties of composites. As shown in Figure 15a, the ultimate tensile strength (UTS) of the composites increases gradually with the increasing drawing reductions. However, this is contrary to the variation trend of elongation (See Figure 15b). When the drawing reduction is 99.7%, the UTS of the composite is up to 581.4 MPa and the elongation is 3.5%. Of note, the composite can be continuously deformed from a diameter of 3.5 mm (as-extruded composite) to an ultra-fine wire with a diameter of 0.195 mm. Under the continuous and severe drawing deformation, a strong and ultra-fine graphene/Cu nanocomposite wire can be achieved, indicating the excellent plastic deformation performance of the as-obtained composite. The underlying reasons for the plastic deformation performance are as follows: (i) Graphene can be uniformly dispersed in the composite due to the in situ grown graphene on Cu powders. During the plastic deformation, the well-dispersibility of graphene can reduce the stress concentration caused by the agglomerated graphene. In Figure 10, the KAM maps reveal that the microstrain distribution in the composite is uniform during the drawing process. Additionally, the homogeneity of microstructure (see Figure 6f) can also improve the uniform plastic deformation ability of the composite. (ii) As previously discussed, graphene can be exfoliated under shear strain during the drawing process, which can act as a lubricant to relax the stress and coordinate the plastic deformation between the grains. Similar results, such as some interfacial reaction products with low melting point can effectively reduce the deforming resistance and improve the deformation ability of composites [55,56]. (iii) The formation of recrystallization can release deformation storage energy, relax internal stress, and reduce deformation resistance, and thus can promote continuous deformation [46]. The improvement of strength of the as-drawn composite wire can be attributed to the thermal mismatch, grain-size refinement, load transfer, and Orowan strengthening reported by the commonly used mechanism [11,57,58]. Additionally, the formation of strong fiber texture can lead to texture strengthening of the composite.

**Figure 16:**
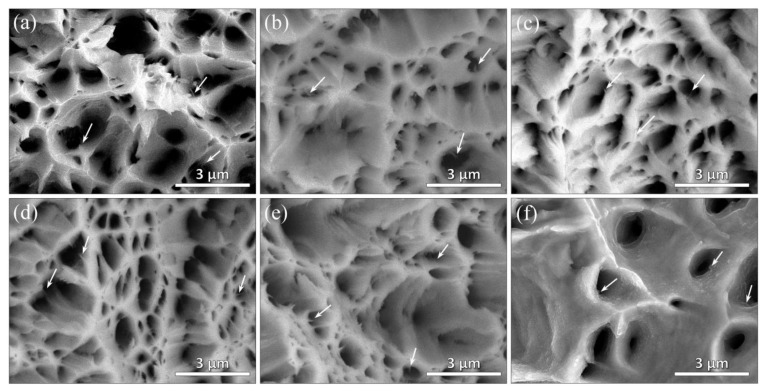
Fracture surface of the composites with different drawing reductions: (**a**) 36.0%, (**b**) 67.3%, (**c**) 73.6%, (**d**) 81.6%, (**e**) 91.3%, (**f**) 99.7%.

Figure 15c shows the electrical conductivity (EC) of the composites with different drawing reductions. It can be found that the EC of composite decreases gradually with the increasing drawing reductions. The EC of the as-drawn composite wire is 45.9 × 10^6^ S/m when the drawing reduction is 99.7%, indicating that the as-drawn composite still has good electrical conductivity. There are complicated factors affecting the EC of the as-drawn composites. First, the increasing number of interfaces resulting from the refinement of grain size and graphene lead to the intensification of electron scattering [59], which will weaken the EC of the composite. Moreover, during the drawing deformation, the formation of dislocations and vacancies in the composite will also increase electron scattering. Nevertheless, the aligned graphene in axis and as-exfoliated graphene can enhance the EC of the composites due to the in-plane orientation of graphene. In addition, the thinned graphene has high EC [60]. Therefore, the EC of composite is a complicated and synergistic effect of the aforementioned factors. Tensile fracture morphology of the as-drawn composites show that no agglomerated graphene can be found on the fracture (see Figure 16). Of note, the size of graphene observed on the fracture surface decreases gradually with the increasing drawing reductions, indicating a good agreement with the observation in Figure 5. Moreover, no pull-out graphene can be found on the fracture, indicating a well-bonding interface of the composites. This can promote a load transfer, which is effective from the matrix to graphene during the tensile process, and obtain the high strength of the composite.

## 4. Conclusions

In this work, the cold drawing behavior of in situ grown graphene/Cu nanocomposite, including the evolution of microstructure, graphene, texture, and properties, have been systematically investigated. Graphene sheets were homogeneously distributed in Cu matrix during the cold drawing process. Furthermore, the refinement and exfoliation of graphene could be clearly found with the increasing drawing reductions. The grain of the composite could be continuously refined to nanoscale and the homogeneity of microstructure was improved after severe drawing deformation. In addition, recrystallization of composites after 80% reduction can be formed, which was affected by a synergistic effect of matrix and graphene. The final fiber texture of the as-drawn composite was a mixture of fiber textures with dominated <111> and minor <100> fiber textures. After cold drawing, the composites presented an enhancement of tensile strength and showed excellent electrical conductivity.

## Figures and Tables

**Table 1:** The diameter and drawing reduction of the composites.

Samples	Diameter (mm)	Drawing Reduction (%)
As-extruded composite	3.5	0
As-drawn composite	2.8	36.0
	2.0	67.3
	1.8	73.6
	1.5	81.6
	1.033	91.3
	0.195	99.7

## Data Availability

Data are contained within the article or Supplementary Material.

## Supplementary Materials

The following supporting information can be downloaded at: https://www.mdpi.com/article/10.3390/nano12050807/s1. Figure S1: XRD patterns of the powders and composites with different drawing reductions. Figure S2: Grain boundaries distribution of the (a) as-sintered, (b) as-extruded, and as-drawn composites with different drawing reductions. Figure S3: Pole figure and inverse pole figure of the as-extruded composite, ED indicates the extrusion direction.
