# Supplementary material for: Microstructure Evolution and Properties Induced by Multi-Pass Drawing of Graphene/Copper Nanocomposite
Source: Nanomaterials (Basel). 2022 Feb 28;12(5):807. doi: 10.3390/nano12050807 (PMC8912415; doi:10.3390/nano12050807)
Supplement: Supplementary file 1 [file nanomaterials-12-00807-s001.zip › nanomaterials-1566023-supplementary.pdf]

Supporting Information

Microstructure Evolution and Properties Induced by Multi-Pass Drawing of Graphene/Copper Nanocomposite

Miao Wang [1,†], Jie Sheng [2,*,†], Changsheng Xing [3], Gang Wang [1], Yuanpei Duan [1] and Lidong Wang [3,*]

[1] School of Materials Science and Engineering, Anhui Polytechnic University, Wuhu 241000, China; wangmiao@ahpu.edu.cn (M.W.); gangwang@ahpu.edu.cn (G.W.); dyp@ahpu.edu.cn (Y.D.)
[2] Laboratory for Space Environment and Physical Science, Research Center of Basic Space Science, Harbin Institute of Technology, Harbin 150001, China
[3] School of Materials Science and Engineering, Harbin Institute of Technology, Harbin 150001, China; xingcs7@163.com
* Correspondence: shengjie@hit.edu.cn (J.S.); wld@hit.edu.cn (L.W.)
† These authors contributed equally to this work.

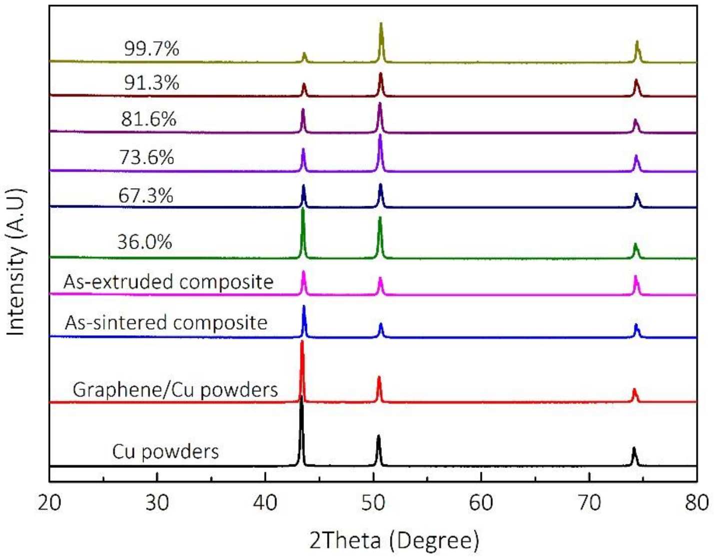

Figure S1. XRD patterns of the powders and composites with different drawing reductions.

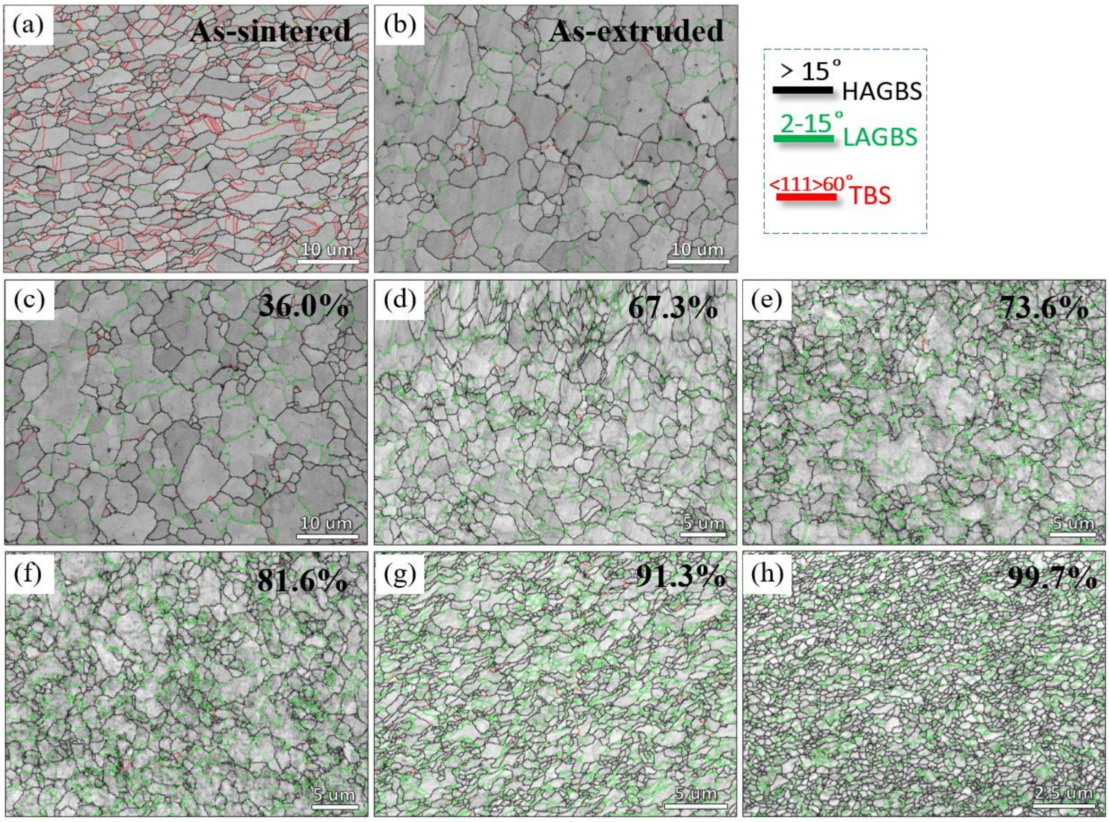

Figure S2. Grain boundaries distribution of the (a) as-sintered, (b) as-extruded and as-drawn composites with different drawing reductions: (c) 36.0%, (d) 67.3%, (e) 73.6%, (f) 81.6%, (g) 91.3% and (h) 99.7%.

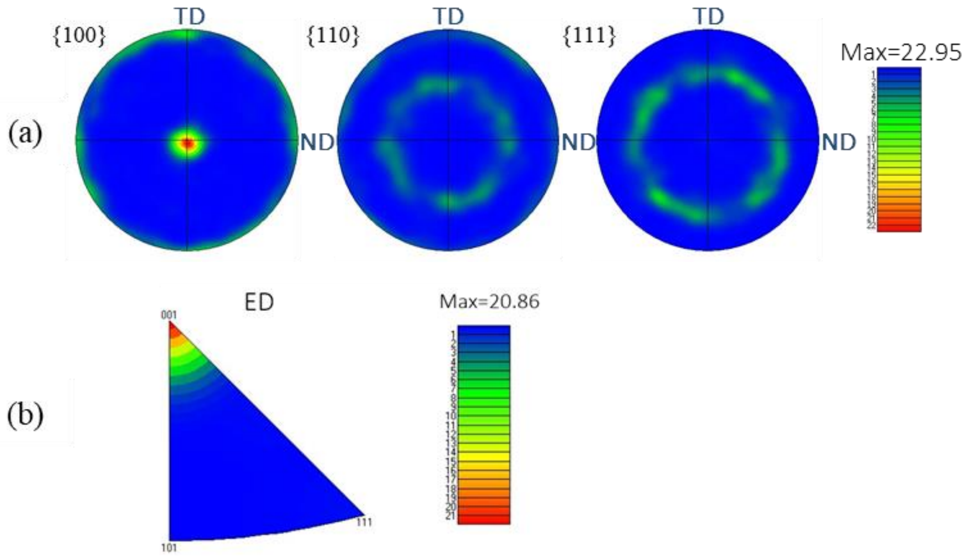

Figure S3. Pole figure (a) and inverse pole (b) figure of the as-extruded composite, ED is the extrusion direction.
